# Rickettsiae in the common pipistrelle *Pipistrellus pipistrellus* (Chiroptera: Vespertilionidae) and the bat soft tick *Argas vespertilionis* (Ixodida: Argasidae)

**DOI:** 10.1186/s13071-020-3885-x

**Published:** 2020-01-09

**Authors:** Shuo Zhao, Meihua Yang, Gang Liu, Sándor Hornok, Shanshan Zhao, Chunli Sang, Wenbo Tan, Yuanzhi Wang

**Affiliations:** 1 School of Medicine, Shihezi University, Shihezi, 832002 Xinjiang Uygur Autonomous Region People’s Republic of China; 2 School of Agriculture, Shihezi University, Shihezi, 832000 Xinjiang Uygur Autonomous Region People’s Republic of China; 3 Department of Parasitology and Zoology, University of Veterinary Medicine, Budapest, Hungary

**Keywords:** *Rickettsia*, Chiroptera, Vespertilionidae, Argasidae

## Abstract

**Background:**

Increasing molecular evidence supports that bats and/or their ectoparasites may harbor vector-borne bacteria, such as bartonellae and borreliae. However, the simultaneous occurrence of rickettsiae in bats and bat ticks has been poorly studied.

**Methods:**

In this study, 54 bat carcasses and their infesting soft ticks (*n* = 67) were collected in Shihezi City, northwestern China. The heart, liver, spleen, lung, kidney, small intestine and large intestine of bats were dissected, followed by DNA extraction. Soft ticks were identified both morphologically and molecularly. All samples were examined for the presence of rickettsiae by amplifying four genetic markers (*17-kDa*, *gltA*, *ompA* and *ompB*).

**Results:**

All bats were identified as *Pipistrellus pipistrellus*, and their ticks as *Argas vespertilionis*. Molecular analyses showed that DNA of *Rickettsia parkeri*, *R. lusitaniae*, *R. slovaca* and *R. raoultii* was present in bat organs/tissues. In addition, nine of the 67 bat soft ticks (13.43%) were positive for *R. raoultii* (*n* = 5) and *R. rickettsii* (*n* = 4). In the phylogenetic analysis, these bat-associated rickettsiae clustered together with conspecific sequences reported from other host and tick species, confirming the above results.

**Conclusions:**

To the best of our knowledge, DNA of *R. parkeri*, *R*. *slovaca* and *R*. *raoultii* was detected for the first time in bat organs/tissues. This is also the first molecular evidence for the presence of *R*. *raoultii* and *R*. *rickettsii* in bat ticks. To our knowledge, *R. parkeri* was not known to occur in Asia. Our results highlight the need to assess rickettsial agents in a broader range of bat species and associated tick species.
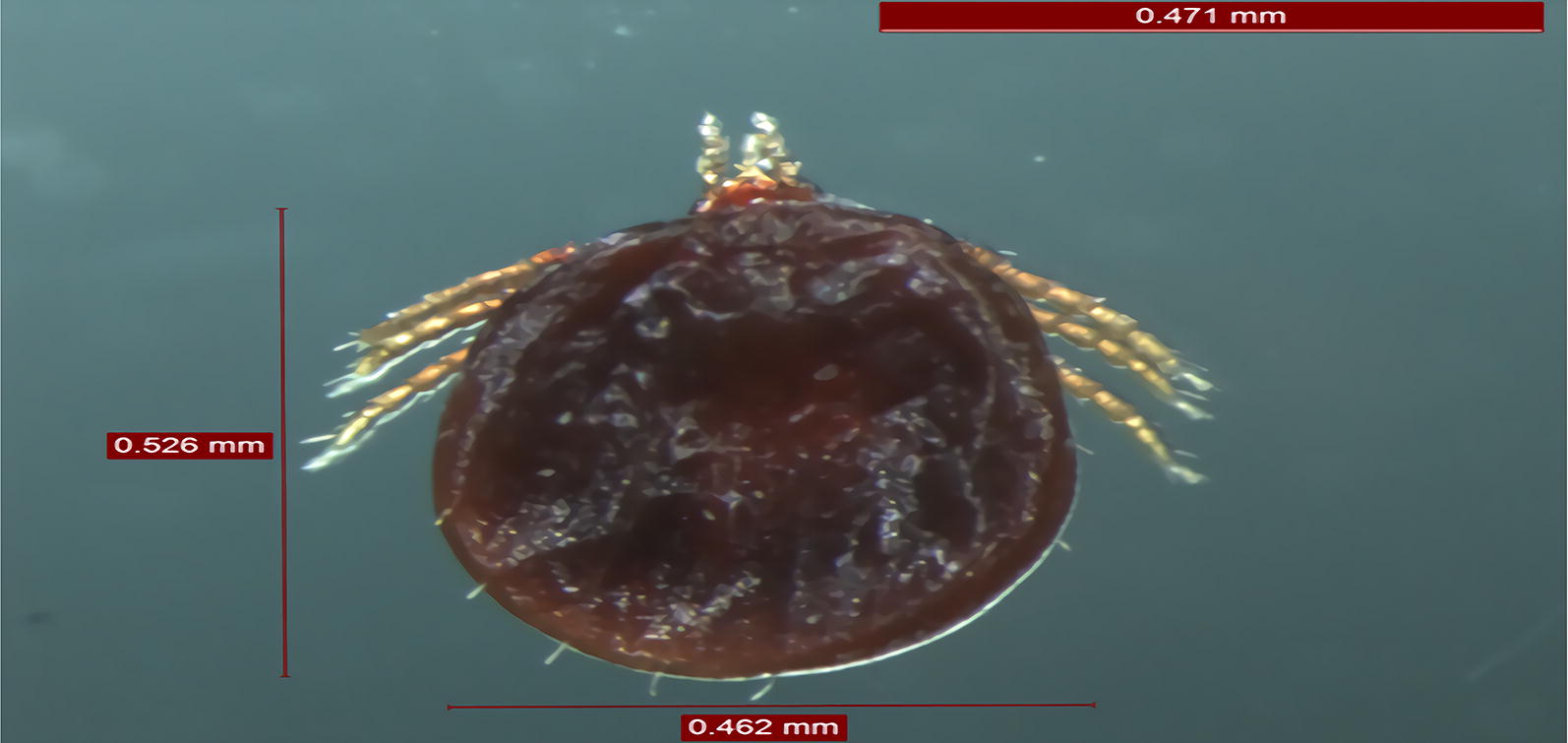

## Background

Bats (order Chiroptera), including at least 1400 species [1], are the only mammals which actively fly [2]. Among the consequences of this trait, bats show a geographically widespread distribution and may even undergo short- to long-distance seasonal migration [2]. Bats are special in their capacity to act as reservoir hosts for intracellular pathogens [3]. Several species of bat-associated soft (Acari: Argasidae) and hard (Acari: Ixodidae) ticks, i.e*. Carios kelleyi*, *Argas vespertilionis*, *A. transgariepinus*, *Ornithodoros* sp., *Ixodes vespertilionis*, *I. ariadnae* and *I. simplex*, were shown to carry DNA of vector-borne bacteria and protozoans [4, 5]. Among these tick species, *A. vespertilionis* (which has a wide distribution in Europe, Africa and Asia) was reported to bite humans [6]. In addition, increasing molecular evidence supports that bats and/or their ticks may harbor vector-borne zoonotic bacteria, such as bartonellae and borreliae [7, 8]. However, the occurrence of rickettsiae in bats and bat ticks appears to be poorly studied, particularly in Eurasia.

Rickettsiae are Gram-negative, obligatory intracellular bacteria, which may cause disease in animals and humans, and are associated with arthropod vectors (such as ticks, lice, fleas or mites), both as transmitters and reservoirs [9]. Concerning rare reports on rickettsiae from bats and their ticks, previously *Rickettsia africae* was detected in the blood of bats in the Caribbean region [10] and several rickettsiae were identified in bat ticks from Central America, Europe, Africa and Asia [11]. However, to the best of our knowledge, a simultaneous analysis of bat tissues and bat ticks for the presence of rickettsiae in central Asia has not been carried out. Our hypothesis is that bats might be susceptible to infection with some rickettsial species. In our study, we aimed at evaluating the susceptibility of bats to rickettsial infection and the involvement of these flying mammals and their ticks in *Rickettsia* transmission cycles in central Asia.

## Methods

### Sample collection and identification

Fifty-four bat carcasses were collected from an idle classroom in Shihezi University, Xinjiang Uygur Autonomous Region (XUAR) in northeastern China during 2015–2019. The heart, liver, spleen, lung, kidney, small intestine and large intestine of bat carcasses were removed, similarly to what has been reported in studies of bat haemoparasites [12]. Genomic DNA was extracted from these organs, as well as from ticks collected from the bats using the 96 Flux Automatic Nucleic Acid Extraction Instrument (Bio Teke, Beijing, People’s Republic of China) with a matching commercial kit (Cell & Tissue Kit, Bio Teke) according to the manufacturer’s instructions. To confirm the morphological identification of bats, the cytochrome *b* (*cytb*) gene was analyzed [13] and a representative *cytb* sequence was deposited in the GenBank database under the accession number MF106222.

Simultaneously, a total of 67 tick larvae were collected from bat bodies. The ticks were morphologically identified according to the standard taxonomic keys as previously described [14]. From five ticks, the *16S* rDNA gene was amplified following a previously reported protocol [15]. The corresponding *16S* rDNA sequence was deposited in the GenBank database under the accession number MF106219.

### Detection of rickettsiae, sequencing and phylogenetic analyses

Four genetic markers, including 17 kDa antigen (*17-kDa*), citrate synthase (*gltA*), and outer membrane proteins A and B (*ompA* and *ompB*) genes were assessed within each sample to investigate the presence of rickettsiae [15]. The primers and PCR cycling conditions in this study are shown in Additional file 1: Text S1 and Table S1. Each PCR assay included a negative control (distilled water instead of tick DNA template) and a positive control (containing sequence-verified DNA from *R. raoultii* obtained from the tick *Dermacentor nuttalli* collected in XUAR) [15]. Purification and sequencing of the PCR products were performed as described above [16, 17].

Sequences were manually edited, aligned and compared to reference GenBank sequences by nucleotide BLASTn program (https://blast.ncbi.nlm.nih.gov). A phylogenetic tree was constructed using the maximum-likelihood and neighbor-joining algorithms implemented in MEGA 6.0 software [18].

## Results

**Fig. 1:**
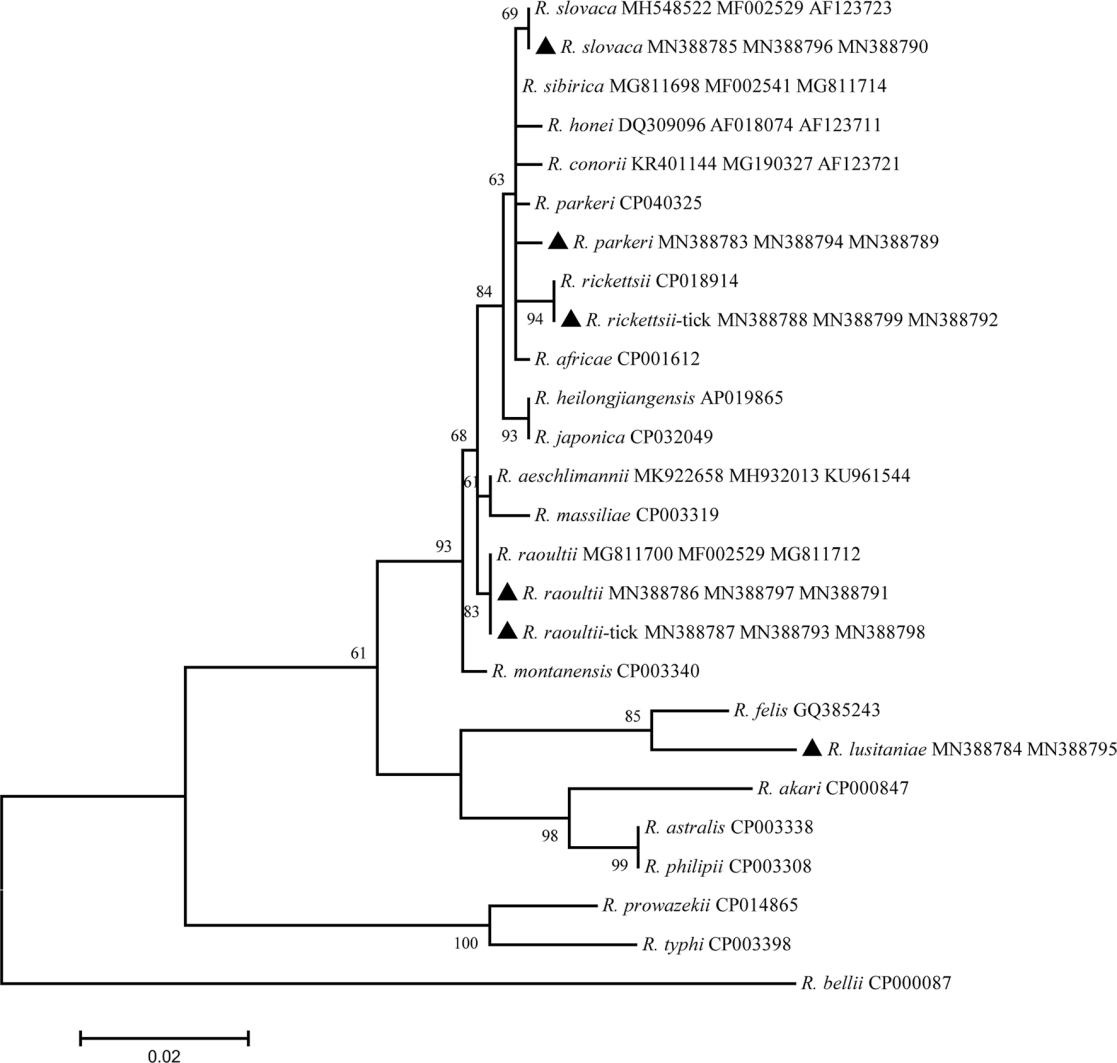
Phylogenetic tree of the *ompA*-*gltA*-*ompB* concatenated sequences for the rickettsial agents in bats and their infesting ticks. The new sequences provided by the present study are indicated by a black triangle

All bats were identified as *Pipistrellus pipistrellus*, and their ticks as *A. vespertilionis*. Out of 378 bat organs/tissues and 67 bat ticks, 6 bats and 9 ticks were positive for the four *Rickettsia* genetic markers (*17-kDa*, *gltA*, *ompA* and *ompB*). Sequencing identified *R. parkeri* in the heart, liver and kidney of a bat, *R. lusitaniae* in the heart, liver and small intestine of a bat, *R. slovaca* in the lung and kidney of two bats and *R. raoultii* was only found in the liver of two bats (Fig. 1, Table 1). Concerning bat ticks, *R. raoultii* was detected in five and *R. rickettsii* in four specimens. Interestingly, four ticks positive to *R*. *raoultii* were removed from a *R*. *raoultii*-positive bat.

**Table 1:** The prevalence of *Rickettsia parkeri*, *R. lusitaniae*, *R*. *slovaca*, *R. raoultii*, *R. rickettsii* detected in the bat organs

Species	Heart	Liver	Spleen	Lung	Kidney	Small intestine	Large intestine
*R. parkeri*	1/54 (1.9%)	1/54 (1.9%)	–	–	1/54 (1.9%)	–	–
*R. lusitaniae*	1/54 (1.9%)	1/54 (1.9%)	–	–	–	1/54 (1.9%)	–
*R. slovaca*	–		–	2/54 (3.7%)	2/54 (3.7%)	–	–
*R. raoultii*	–	2/54 (3.7%)	–	–	–	–	–

Regarding sequence comparisons based on the four genetic markers, *R. parkeri* in this study had sequence identities within the range of 99.7–100% when compared with the sequence of *R. parkeri* from the tick *Amblyomma ovale* in Colombia (GenBank: CP040325); *Rickettsia lusitaniae* showed 98.6–100% identity compared with the sequence of *R. lusitaniae* from *A. vespertilionis* infesting *P. pipistrellus* in China [11]; *Rickettsia slovaca* had 99.8% sequence identity with a conspecific bacteria from *Dermacentor marginatus* in Turkey; *Rickettsia raoultii* showed 99.1–100% identity with *R. raoultii* from *D. nuttalli* infesting *Spermophilus undulatus* in northwestern China [13]; and *Rickettsia rickettsii* was 99.7–99.9% identical to *R. rickettsii* from *D. variabilis* in the USA [19]. The detailed similarities and divergences of the sequences in this study are shown in Table 2 and Additional file 2: Table S2.

**Table 2:** All data for the newly sequenced isolates and their GenBank accession numbers for all genes

Gene	Species	Host	Isolate	GenBank ID
*cytb*	*P. pipistrellus*	*P. pipistrellus*	*P. pipistrellus*	MF106222
*16S*	*A. vespertilionis*	*A. vespertilionis*	*A. vespertilionis*	MF106219
*OmpA*	*R. parkeri*	*P. pipistrellus*	Heart, liver, kidney	MN388783
*OmpA*	*R. lusitaniae*	*P. pipistrellus*	Heart, liver, small intestine	MN388784
*OmpA*	*R. slovaca*	*P. pipistrellus*	Lung, kidney	MN388785
*OmpA*	*R. raoultii*	*P. pipistrellus*	Liver	MN388786
*OmpA*	*R. raoultii*	*A. vespertilionis*	*A. vespertilionis*	MN388787
*OmpA*	*R. rickettsii*	*A. vespertilionis*	*A. vespertilionis*	MN388788
*OmpB*	*R. parkeri*	*P. pipistrellus*	Heart, liver, kidney	MN388789
*OmpB*	*R. slovaca*	*P. pipistrellus*	Lung, kidney	MN388790
*OmpB*	*R. raoultii*	*P. pipistrellus*	Liver	MN388791
*OmpB*	*R. rickettsii*	*A. vespertilionis*	*A. vespertilionis*	MN388792
*OmpB*	*R. raoultii*	*A. vespertilionis*	*A. vespertilionis*	MN388793
*gltA*	*R. parkeri*	*P. pipistrellus*	Heart, liver, kidney	MN388794
*gltA*	*R. lusitaniae*	*P. pipistrellus*	Heart, liver, small intestine	MN388795
*gltA*	*R. slovaca*	*P. pipistrellus*	Lung, kidney	MN388796
*gltA*	*R. raoultii*	*P. pipistrellus*	Liver	MN388797
*gltA*	*R. raoultii*	*A. vespertilionis*	*A. vespertilionis*	MN388798
*gltA*	*R. rickettsii*	*A. vespertilionis*	*A. vespertilionis*	MN388799
*17-kDa*	*Rickettsia* sp.	*P. pipistrellus*	*P. pipistrellus*	MN388800

## Discussion

Based on a recent review, *R. lusitaniae*, *R. slovaca*, *R. raoultii* and *R. rickettsii* have already been found to occur in Asia [20]. To the best of our knowledge, *R. parkeri* was detected for the first time on the Eurasian continent in the present study. Previously, *R. conorii* and *R. orientalis* have been identified in human urine and urine of albino Swiss mice [21, 22] and *R. helvetica* has been detected in bat feces [23]. Here, the kidneys from common pipistrelle bats were positive for *R. parkeri* and *R. slovaca*, while the small intestine was positive for *R. lusitaniae*, and the lung was positive for *R*. *slovaca*. These findings suggest that *P. pipistrellus* might become infected with *R. parkeri*, *R. lusitaniae* and *R. slovaca*. Importantly, PCR-positivity of the kidneys and the small intestine warrant further studies to investigate, if rickettsiae also pass with the faeces and urine of bats, because some of the rickettsiae (including *R. rickettsii* detected here in bat ticks) are known to cause infection *via* aerosol transmission [24].

In our previous studies we provided molecular evidence for the presence of *R*. *raoultii* in road-killed marbled polecat (*Vormela peregusna*) and its infesting tick *Haemaphysalis erinacei* [18]. Here, we detected *R. raoultii* in two bat livers and bat ticks for the first time. Interestingly, the *gltA, ompA* and *ompB* sequences from bat tissues were 100% identical with those from the PCR-positive bat ticks. *Argas vespertilionis* has a broad geographical distribution in the Old World, parasitizing several bat species, such as *Eptesicus serotinus* and *P. pipistrellus* [25, 26]. Thus, the present findings suggest that, in relevant regions of Eurasia, *R. raoultii* may co-circulate between the bat *P. pipistrellus* and the bat tick *A. vespertilionis*.

In 1994, Jaenson et al. [27] reported that two persons living near Stockholm were bitten by the bat tick *A. vespertilionis* in their bedroom, and consequently clinical signs (fever, ulceration, erythema and edema) developed. It has also been reported that certain soft tick species can be infected and are capable of transmitting human pathogenic rickettsiae, as exemplified by *R. slovaca* and *R. rickettsii* in *Argas persicus* and *Ornithodoros* spp., respectively [28, 29]. These literature data underline the significance of the present findings and justify the need to evaluate further the actual epidemiological risks associated with the presence of *R*. *raoultii*, *R*. *slovaca*, *R. parkeri* and *R*. *rickettsii* in bats and their ticks.

Bats are susceptible to several vector-borne disease agents, including *Trypanosoma cruzi*, *Babesia vesperuginis* and *Polychromophilus murinus* [30]. Furthermore, some of these microorganisms may cause pathological changes in bats, as exemplified by *B. vesperuginis*, inducing anemia, splenomegaly, hemoglobinuria, and elevated reticulocyte and leukocyte counts [31]. In addition, the pathogenic role of rickettsiae is documented in several mammalian species (e.g. in the pine vole, *Microtus pinetorum*, these bacteria elicit tremor, fur ruffling and heavy breathing [32]). However, the clinico-pathological role of rickettsial infection in bats remains to be elucidated.

Here, 11.11% of bats (6/54) were infected with *R. parkeri*, *R. lusitaniae*, *R. raoultii* or *R. slovaca.* In addition, bat soft ticks contained the DNA of *R. raoultii* and *R. rickettsii*. These results warrant future studies, investigating (i) the routes of infection for bats (i.e. whether bat soft ticks are competent vectors or not; and if so, whether rickettsiae are transmitted by them transstadially and/or transovarially); (ii) clinical and pathological aspects of rickettsial infection in bats; as well as (iii) epidemiological risks (if any) of zoonotic transmission.

## Conclusions

To our knowledge, this study provides the first report of *R. parkeri*, *R*. *slovaca* and *R*. *raoultii* in bats. *Rickettsia raoultii* and *R. rickettsii* were detected for the first time in bat soft ticks. Our findings contribute to the knowledge on the geographical distribution, and tick and vertebrate hosts for rickettsiae.

## Supplementary information

**Additional file 1: Text S1.** PCR protocol for the detection of *Rickettsia* spp. in bats and their ticks. **Table S1.** Primers used for the identification of *Rickettsia* spp.

**Additional file 2: Table S2.** Closest sequences to the partial *17-kDa*, *gltA*, *ompA*, *ompB* gene sequences of *Rickettsia parkeri*, *R. lusitaniae*, *R*. *slovaca*, *R. raoultii*, *R. rickettsii* detected in bats and their ticks in the present study.

## Data Availability

Data supporting the conclusions of this article are included within the article and its additional files. The newly generated sequences were submitted to the GenBank database under the accession numbers MF106222, MF106219 and MN388783-MN388800.

## Abbreviations

SFG: spotted fever group
TG: typhus group
*cytb*: cytochrome *b*
*17-kDa*: 17 kDA antigen
*gltA*: citrate synthase
*ompA*: outer membrane proteins A
*ompB*: outer membrane proteins B
XUAR: Xinjiang Uygur Autonomous Region
PCR: polymerase chain reaction
