# Supplementary material for: Rickettsiae in the common pipistrelle Pipistrellus pipistrellus (Chiroptera: Vespertilionidae) and the bat soft tick Argas vespertilionis (Ixodida: Argasidae)
Source: Parasit Vectors. 2020 Jan 9;13:10. doi: 10.1186/s13071-020-3885-x (PMC6953312; doi:10.1186/s13071-020-3885-x)
Supplement: Supplementary file 1 — Additional file 1: Text S1. PCR protocol for the detection of Rickettsia spp. in bats and their ticks. Table S1. Primers used for the identification of Rickettsia spp. [file 13071_2020_3885_MOESM1_ESM.docx]

**Additional file 1: Text S1.**

**PCR protocol for the detection of *Rickettsia* spp. in bats and their ticks.**

The PCR equipment was a TechneTC-412 thermal cycler, Barloworld Scientific, Cambridge, UK.

**1. PCR amplification of four gene markers (*17-kDa*, *gltA*, *ompA*, *ompB*) sequences from ticks**

Each reaction consisted of 1 μL of tick genomic DNA (50 ng) and 12.5 μL of a PCR mix containing 50 mM KCl, 10 mM Tris-HCl (pH 8.3), 1.5 mM MgCl_2_, 250 μM of each dNTP, 40 pmol of each primer (*17-kDa, gltA, ompA, ompB*), and 1.0 U of Taq DNA polymerase (TaKaRa Taq Version 2.0, Takara, Dalian, China).

**2.PCR cycling conditions of four Rickettsia gene markers (*17-kDa*, *gltA*, *ompA*, *ompB*)**

The PCR cycling conditions consisted of a pre-PCR of 95 ℃ for 5 min, followed by 35 cycles of 95 ℃ 40 s, annealing for 40 s at 61℃ for *17-kDa*, 50℃for *ompA*-out and *gltA*-out, 59 ℃ for *ompA*-in and *gltA*-in, 56 ℃ for *ompB*, and an extension of 72 ℃ for 1min, with a final extension of 72 ℃ for 10 min.

**Table S1.** Primers used for the identification of *Rickettsia* spp.

| Gene | Primer | Sequence（5'to3'） | Reference |
| --- | --- | --- | --- |
| *17-kDa* | *17kDa*-out1 | GCTTTACAAAATTCTAAAAACCATATA | [1] |
|  | *17kDa*-out2 | TGTCTATCAATTCACAACTTGCCGTT |  |
|  | *17kDa*-in1 | GCTCTTGCAACTTCTATGTT |  |
|  | *17kDa*-in2 | CATTGTTCGTCAGGTTGGCG |  |
| *ompA* | *ompA*-out1 | ATGGCGAATATTTCTCCAAAA | [1] |
|  | *ompA*-out2 | AGTGCAGCATTCGCTCCCCCT |  |
|  | *ompA*-in1 | CTTAAAGCCGCTTTATTCACCACCTC | [1] |
|  | *ompA*-in2 | CCTGTATAATTATCGGCAGGAGC |  |
| *gltA* | *gltA*-out1 | ATGACCAATGAAAATAATAAT | [1] |
|  | *gltA*-out2 | ATTGCAAAAAGTACAGTGAACA |  |
|  | *gltA*-in1 | GGAATCTTGCGGCATCGAGGATATG | [1] |
|  | *gltA*-in2 | CCATAGCTTTATAGATAATACCCG |  |
| *ompB* | *ompB*-out1 | ACAGCTACCATAGTAGCCAG | [1] |
|  | *ompB*-out2 | TGCAGTATAGTTACCACCG |  |
|  | *ompB*-in1 | TGCTGCGGCTTCTACATT |  |
|  | *ompB*-in2 | ACCGCCAGCGTTCCCTAT |  |

**References**

1. Zhao S, Yang M, Jiang M, Yan B, Zhao S, Yuan W, et al. *Rickettsia raoultii* and *Rickettsia sibirica* in ticks from the long-tailed ground squirrel near the China–Kazakhstan border. Exp Appl Acarol. 2019;77:425-33
