# Supplementary material for: Rickettsiae in the common pipistrelle Pipistrellus pipistrellus (Chiroptera: Vespertilionidae) and the bat soft tick Argas vespertilionis (Ixodida: Argasidae)
Source: Parasit Vectors. 2020 Jan 9;13:10. doi: 10.1186/s13071-020-3885-x (PMC6953312; doi:10.1186/s13071-020-3885-x)
Supplement: Supplementary file 2 — Additional file 2: Table S2. Closest sequences to the partial 17-kDa, gltA, ompA, ompB gene sequences of Rickettsia parkeri, R. lusitaniae, R. slovaca, R. raoultii, R. rickettsii detected in bats and their ticks in the present study. [file 13071_2020_3885_MOESM2_ESM.docx]

**Additional file 2: Table S2.**

Closest sequences to the partial *17-kDa*, *gltA*, *ompA*, *ompB* genes, sequences of the *Rickettsia parkeri*, *R.* *lusitaniae*, *R*. *slovaca, R. raoultii, R. rickettsii* detected in bats and their ticks in the present study.

| *Rickettsia* species | Host/tick | Closest relative sequences to the genes in GenBank [bp/bp (%)] | Accession number (present study) | Closest match sequence  accession number | Reference |
| --- | --- | --- | --- | --- | --- |
| *Rickettsia parkeri* | *Pipistrellus pipistrellus* | *OmpA*: 446/446(100) | MN388783 | CP040325 | - |
|  |  | *gltA*: 928/931(99.7) | MN388794 | CP040325 | - |
|  |  | *OmpB*: 1063/1063(100) | MN388789 | CP040325 | - |
| *Rickettsia* *lusitaniae* | *Pipistrellus pipistrellus* | *OmpA*: 437/443(98.6) | MN388784 | MH383149 | 1 |
|  |  | *gltA*: 705/706(99.9) | MN388795 | MH383142 | 1 |
|  |  | *17KD*: 393/393(100) | MN388800 | MH383145 | 1 |
| *Rickettsia* *slovaca* | *Pipistrellus pipistrellus* | *OmpA*: 445/446(99.8) | MN388785 | MH548522 | - |
|  |  | *gltA*: 931/931(100) | MN388796 | MF002529 | - |
|  |  | *OmpB*: 1062/1063(99.9) | MN388790 | AF123723 | - |
| *Rickettsia raoultii* | *Pipistrellus pipistrellus* | *OmpA*: 439/443(99.1) | MN388786 | MG811700 | 2 |
|  |  | *gltA*: 931/931(100) | MN388797 | MF002529 | - |
|  |  | *OmpB*: 1063/1063(100) | MN388791 | MG811712 | 2 |
|  | *Argas vespertilionis* | *OmpA*: *441/443(99.5)* | MN388787 | MG811700 | 2 |
|  |  | *gltA: 931/931(100)* | MN388793 | MG811710 | 2 |
|  |  | *OmpB*: 1063/1063(100) | MN388798 | MG811712 | 2 |
| *Rickettsia rickettsii* | *Argas vespertilionis* | *OmpA*: 445/446(99.8) | MN388788 | CP018914 | 4 |
|  |  | *gltA:* 928/931(99.7) | MN388799 | CP018914 | 4 |
|  |  | *OmpB*: 1062/1063(99.9) | MN388792 | CP018914 | 4 |

**References**

1. Hornok S, Szőke K, Meli ML, Sándor AD, Görföl T, Estók P et al. Molecular detection of vector-borne bacteria in bat ticks (Acari: Ixodidae, Argasidae) from eight countries of the Old and New Worlds. Parasit Vectors. 2019;12:50.
2. Zhao S, Yang M, Jiang M, Yan B, Zhao S, Yuan W, et al. Rickettsia raoultii and Rickettsia sibirica in ticks from the long-tailed ground squirrel near the China–Kazakhstan border. Exp Appl Acarol. 2019;77:425-33
3. Milhano N, Palma M, Marcili A, Núncio MS, de Carvalho IL, de Sousa R. *Rickettsia lusitaniae* sp. nov. isolated from the soft tick *Ornithodoros erraticus* (Acarina: Argasidae). Comp Immunol Microbiol Infect Dis. 2014;37:189–93.
4. Noriea NF, Clark TR, Mead D, Hackstadt T. Proteolytic cleavage of the immunodominant outer membrane protein rOmpA in *Rickettsia rickettsii*. J Bacteriol. 2017;199:e00826.
